# Compact akinetic swept source optical coherence tomography angiography at 1060 nm supporting a wide field of view and adaptive optics imaging modes of the posterior eye

**DOI:** 10.1364/BOE.9.001871

**Published:** 2018-03-26

**Authors:** Matthias Salas, Marco Augustin, Franz Felberer, Andreas Wartak, Marie Laslandes, Laurin Ginner, Michael Niederleithner, Jason Ensher, Michael P. Minneman, Rainer A. Leitgeb, Wolfgang Drexler, Xavier Levecq, Ursula Schmidt-Erfurth, Michael Pircher

**Affiliations:** 1 Center for Medical Physics and Biomedical Engineering, Medical University of Vienna, Vienna, Austria; 2 Imagine Eyes, Orsay, France; 3 Department of Ophthalmology and Optometry, Medical University of Vienna, Vienna, Austria; 4 Christian Doppler Laboratory for Innovative Optical Imaging and Its Translation to Medicine, Medical University of Vienna, Vienna, Austria; 5 INSIGHT Photonic Solutions, Inc., Lafayette, CO, USA

**Keywords:** (110.4500) Optical coherence tomography, (170.3890) Medical optics instrumentation, (170.4470) Ophthalmology, (110.1080) Active or adaptive optics

## Abstract

Imaging of the human retina with high resolution is an essential step towards improved diagnosis and treatment control. In this paper, we introduce a compact, clinically user-friendly instrument based on swept source optical coherence tomography (SS-OCT). A key feature of the system is the realization of two different operation modes. The first operation mode is similar to conventional OCT imaging and provides large field of view (FoV) images (up to 45° × 30°) of the human retina and choroid with standard resolution. The second operation mode enables it to optically zoom into regions of interest with high transverse resolution using adaptive optics (AO). The FoV of this second operation mode (AO-OCT mode) is 3.0° × 2.8° and enables the visualization of individual retinal cells such as cone photoreceptors or choriocapillaris. The OCT engine is based on an akinetic swept source at 1060 nm and provides an A-scan rate of 200 kHz. Structural as well as angiographic information can be retrieved from the retina and choroid in both operational modes. The capabilities of the prototype are demonstrated in healthy and diseased eyes.

## 1. Introduction

Optical coherence tomography (OCT) has shown to be an essential tool for detecting and investigating retinal diseases [1]. Driven by clinical needs, OCT technology was rapidly transferred into clinics and is now regarded as a standard imaging technology in eye care [2]. Recently, capabilities of OCT for diagnosis have been further enhanced by the implementation of OCT angiography (OCTA) [3–7]. However, underlying mechanisms of diseases such as age related macular degeneration (AMD) [8, 9] or diabetic retinopathy (DR) [10] are still poorly understood. In order to get more insight into these processes, information that is obtained at a cellular level in the living human retina is required. Commercial OCT instruments lack sufficient transverse resolution for cellular imaging because aberrations introduced to the imaging beam prevent the use of the full numerical aperture of the eye. Thus, imaging at a cellular level will be limited and individual cells such as cone photoreceptors can only be visualized under specific conditions in healthy volunteers [11, 12]. In order to overcome this limitation, adaptive optics (AO) has been combined with OCT [13, 14]. Meanwhile a couple of research grade instruments have been presented and overviews of this technique can be found elsewhere [15–18]. AO-OCT enables the visualization of various cell types in the retina such as cone photoreceptors [19–21], rod photoreceptors [22], retinal pigment epithelium cells [22–24], erythrocytes [25] or even ganglion cells [26].

One specific challenge for translating these systems into clinics is the rather bulky size of the instruments. In order to address this point more compact AO-OCT prototypes have been presented [27–30]. However, all these compact systems are operated in the 750-850nm wavelength range and use, apart from recently introduced wavefront sensor less systems [31, 32], spectral domain OCT (i.e. they are based on a spectrometer in the detection arm). For such systems the limited spectral resolution causes the system sensitivity to decay rapidly with imaging depth, which sets high demands on patient alignment as the retina needs to be imaged close to zero delay in order to achieve highest sensitivity. In addition, for high speed systems, the readout time of the camera takes a significant amount of time in comparison to the exposure time which results in a reduced duty cycle within an A-scan. The latter limitation has been overcome by implementing optical switches in combination with four spectrometers [33]. However, the additional costs, synchronization efforts and space requirements are not negligible.

Common to all AO-OCT systems is that they can be operated only with a rather small scanning angle resulting in a small field of view (FoV) on the retina. This is mainly due to the limited isoplanatic angle in the eye [34]. Only within this angle the introduced aberrations can be assumed as constant across the field of view and can be corrected for. Associated with the small field of view are difficulties in finding regions of interest and to determine the imaged area on images recorded with complimentary state of the art imaging technologies. Thus, it is quite common to combine AO-OCT with other imaging modalities in a single instrument [27, 28, 30, 35].

In this paper we present a different approach in order to address the issues mentioned above. To reduce the sensitivity roll off with depth the compact AO-OCT instrument is based on swept source technology with long coherence length operating in the 1060nm wavelength band. In addition, a phase stable akinetic swept source [36] is used that allows exploiting the phase information of the light for generating angiographic data of the retina or for Doppler OCT without the need of additional phase correcting procedures. Difficulties associated with the limited FoV of AO-OCT are addressed with the implementation of two imaging modes. The first mode by passes the AO imaging path and provides overview OCT images with standard resolution. The second mode enables to optically zoom into regions of interest with high transverse resolution and adaptive optics correction. The entire instrument has a similar compactness as commercial OCT instruments and enables an easy translation into clinical settings. The performance of the system is tested by imaging healthy and diseased eyes in vivo.

## 2. Methods

### Imaging system

Figure 1

**Fig. 1:**
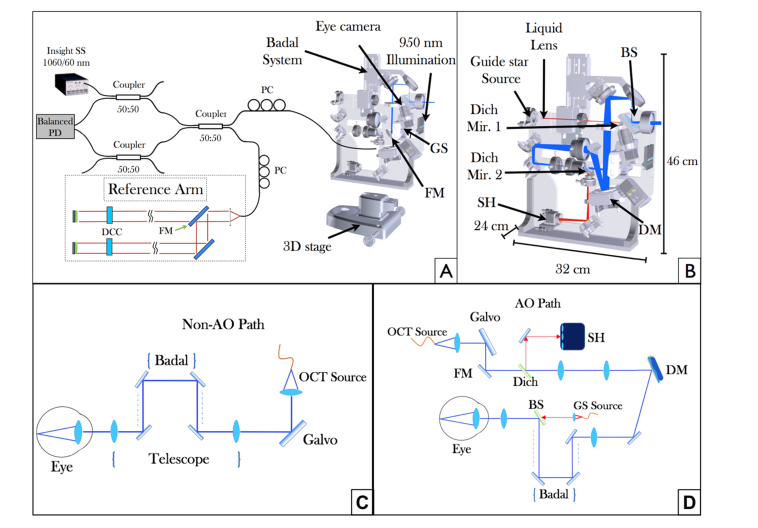
Overview of the compact swept source (SS)-OCT prototype system. (A) OCT interferometer configuration and beam propagation in the large field of view (FoV) imaging mode. PC polarization controller, PD photodiode, GS galvanometer scanner, FM flip mirror, DCC dispersion compensation component. (B) Scheme of the sample arm optics showing the beam propagation in AO imaging mode. The blue line shows the optical path of the OCT beam, the red line represents the light path of a guide star used for wavefront sensing. The dimensions of the sample arm housing are also specified. (C) Detailed beam propagation for the non-AO imaging mode. (D) Detailed beam propagation for the AO-imaging mode. Dich Mir. 1,2 dichroic mirrors, BS beam splitter, DM deformable mirror, SH Shack Hartmann wavefront sensor.

shows a schematic drawing of the system. The OCT engine is based on a 1060 nm akinetic swept source (Insight Photonic Solutions, Inc., Lafayette, Colorado, USA) with an A-scan rate of 200 kHz. The sweep range of the laser is 60nm with a rectangular spectral shape. To reduce the visible side-lobes in the coherence function originating from this shape we performed spectral shaping that resulted in a measured axial resolution (full width at half maximum (FWHM) of the coherence function) of 10.2µm in air. This translates to ~7.3µm in tissue considering a refractive index of 1.4. The (single path) coherence length (determined by the instantaneous linewidth) of the laser is >>16mm. Phase jitter between A-scans is very small and similar to a system presented in [37]. In order to quantify this jitter we performed phase measurements on the interference signal between front- and back-surface of a glass plate (common path interference) and found a FWHM of the resulting Gaussian phase distribution of 3mrad. Because of the high signal to noise ratio that could be achieved in this sample, the value is an order of magnitude smaller than corresponding phase measurements with spectral domain OCT in a scattering sample [38]. Thus, phase noise introduced by the light source can be neglected and there is no need of additional instrumentation for phase stabilization which simplifies the overall instrument. For OCT imaging a symmetrical Michelson interferometer [39] is used based on three 50:50 fiber couplers (wavelength Flattened Coupler, Gould, Millersville, USA), which were spliced together to reduce losses. Polarization paddles in the sample and reference arm are used to match the polarization state of the light returning from both arms, respectively.

The sample arm of the prototype (cf. Fig. 1(A)) is mounted on a 3D stage and can be moved in three dimensions in order to align the instrument in respect to the subject’s eye. The prototype incorporates two different OCT imaging modes. The large FoV mode (cf. Fig. 1(C)) covers an area of up to 45° × 30° on the retina while the high transverse resolution AO-OCT mode (cf. Fig. 1(D)) covers an area of 3° × 2.8° on the retina. Both modes use the same pair of galvanometer scanners and switching between the two modes is achieved with two flip mirrors that are located in the sample and reference arm, respectively.

In the large FoV mode, the beam exiting the fiber coupler of the sample arm is collimated and sent to the galvanometer scanners (cf. Fig. 1(C)). A telescope images the pivot point of the scanners into the pupil plane of the eye. To account for varying ametropia of subjects a Badal system is implemented into the telescope which allows for changing the collimation of the beam exiting the system. The telescope reduces the diameter of the imaging beam which results in 0.6 mm at the pupil of the eye for this operation mode. This is translated by the optics of the eye to a transverse resolution of ~30µm on the retina [18]. The light that is backscattered from the retina traverses the same path back and is coupled into the single mode fiber of the OCT interferometer.

The interference signal is detected at the interferometer exit by a balanced photodetector (PDB130C, Thorlabs Inc., NJ, USA) and sampled by a 12-bit A/D digitizer (data acquisition (DAQ) card, Alazartech Technologies, Inc., Canada, ATS9350). The k- clock provided by the swept source is used to synchronize the acquisition of the DAQ card with a delay on the start of the sweep in order to remove invalid data points [36]. These points were determined in a calibration measurement using a mirror at the end of the sample arm of the interferometer. After Fourier Transformation of the interference signal, a total number of 780 pixels in depth are obtained for each A-scan (without zero-padding). The driving signal for the galvanometer scanners is generated with a field programmable gate array FPGA (NI PCIe-7852R, National Instruments, Austin, USA). The driving voltage for the x-scanner (fast axis) is changed stepwise whenever the FPGA receives an A-scan trigger from the light source. The drive shape of the scanner is a saw-tooth pattern with an exponential decay of the voltage for the backward scan that takes approximately1/10 of the time of the forward scan. After a specified number of A-scans is reached a B-scan trigger is generated and the driving voltage value is updated for the y-scanner (slow axis). The scanners (Cambridge Technologies) are operated with a mechanical scanning angle of 2-4° with a clear aperture of 5-8mm.

When switched to the high resolution AO-OCT mode, the beam reflected by the scanners is diverted by the flip mirror and traverses a different telescope (cf. blue light path in Fig. 1(B)). This telescope enlarges the beam diameter to match the size of the deformable mirror (DM) and images the pivot point of the scanners onto the DM (Mirao52-e RC, Imagine Eyes, Orsay, France). The DM directs the light to a second telescope which is the same as for the large FoV mode and contains the Badal system. Similar as in the other imaging mode, the beam diameter is reduced by this telescope. As the entrance beam diameter is of the size of the DM, the beam diameter is reduced to 6 mm at the pupil plane of the eye resulting in a ~3µm spot diameter on the retina.

For AO correction, a super luminescent diode (SLD) (Exalos, Schlieren, Switzerland) is used at 750 nm to produce a guide star (~25-30µm spot size) on the retina (cf. red path in Fig. 1(B)). This specific wavelength region was chosen in order to enable a clear separation between imaging and wavefront sensing beam via dichroic mirrors. The light from the SLD exits a single mode fiber and passes through a liquid lens. The refractive power of the lens can be changed in order to account for different eye lengths. The plane of the lens is conjugated optically to the pupil plane of the eye via a telescope. The light of the guide star is coupled with the imaging beam via a beam splitter (90% reflecting, 10% transmitting) within the last telescope. Thus, both beam paths share the same last lens of the telescope. After being backscattered at the retina, the light at both wavelength regions traverses all elements of the AO imaging path. Before the entrance collimator of the AO-OCT imaging beam, a dichroic mirror (Dich Mir. 2 in Fig. 1(B)) is placed such that it diverts the light of the guide star onto the Shack-Hartmann wavefront sensor (Haso first, Imagine Optics, Orsay, France) for wavefront measurements. The AO correction runs in a closed loop at 10 Hz during the entire measurements.

To facilitate the correct positioning of the subject’s eye the instrument is equipped with an eye camera and an anterior segment illumination at 950 nm (LED SFH 4511, OSRAM, Munich, Germany). Multiple sources emit light diffusively and we measured 30µW of power at the location of the eye within an area of 3cm^2^. The light backscattered from the anterior segment traverses the first telescope lens and is reflected by a dichroic mirror (Dich Mir. 1 in Fig. 1(B)) before it is detected by an area camera (Guppy F033, Allied Vision, Stadtroda, Germany). Finally, an internal fixation target is projected onto the retina which allows stable fixation of the imaged subject.

### Imaging protocol

The sampling of the large FoV mode (45°x25°) typically is 1000 A-scans per B-scan and 1000 B-scans along the slow-axis which results with an A-scan rate of 200KHz in an acquisition time of ~5 seconds for the entire volume. As this imaging mode results in a rather low sampling density (~13µm distance between A-scans) we additionally performed measurements with a reduced field of view (medium FoV of 27° × 13°) with the same sampling by simply reducing the voltage that is sent to the galvanometer scanners. In the case of the AO imaging mode the sampling is reduced to 500 A-scans per B-scan and 600 B-scans along the slow axis which results in an acquisition time of ~1.5 seconds. For patients with poor fixation capabilities the sampling is reduced to decrease the measurement time and minimize the appearance of motion artefacts. The power at the eye of the imaging beam is 2 mW for the large FoV mode and 1mW for the AO mode (optical losses are more pronounced for this mode). In addition, for this imaging mode 40 µW of light power is sent to the eye for generation of the guide star. In terms of eye safety only thermal effects need to be considered at the used wavelength region. The overall power that is sent to the eye was below the maximum permissible exposure values given in the laser safety standards [40]. With these light exposures a sensitivity of 97 dB was measured for the large FoV imaging mode and 92 dB for the AO mode. The slightly lower sensitivity compared to instruments operating in the same wavelength region probably results from the specific interferometer design and the power losses within the AO imaging mode. The sensitivity roll-off of the system is mainly determined by the cut-off frequency of the used detectors and is negligible within the first 4 mm. At longer imaging depths, the cut off frequency of the detectors will result in lower sensitivities.

Imaging of subjects was done according to the tenets of the Declaration of Helsinki and under a protocol that was approved by the local ethics committee of the Medical University of Vienna (EK Nr: 1631/2016). Prior to the measurements informed consent was obtained from all subjects after explaining the form and nature of the measurements. Healthy volunteers and patients were selected by the Department of Ophthalmology of the Medical University of Vienna. Selection criteria were the ability to fixate and negligible media opacities. In total, 10 subjects participated in the study with an age range between 28 and 82 years. For patient imaging the pupils were artificially dilated by administering eye drops (Tropicamid 1%) prior to each imaging session. This procedure was not necessary for healthy volunteers as the natural pupil in the dark measurement environment was sufficiently large (larger than the beam diameter exiting the system).

A typical imaging session started with recording of an OCT volume centered at the fovea in the large FoV mode. The recorded data was immediately processed and a pseudo fundus image was generated. This allowed for selection of regions of interest for the AO imaging mode. In order to acquire a high quality B-scan of an interesting location, a horizontal line on the pseudo fundus image was selected and 100 B-scans at this position were recorded and averaged.

For OCT angiography, the sampling of the data was reduced to 1000 A-scans per B-scan and 500 B-scans along the slow axis. The recording time of a volume was therefore increased by a factor of two (i.e. ten seconds) because at each B-scan location four images were recorded. After recording of angiographic data the position of the flip mirrors was changed to enable the AO mode. The fixation target was re-positioned in order to image the selected region of interest. The light for the guide star was switched on and the AO loop was closed. After a small residual wavefront error (small root mean square (RMS) value) had been achieved, several AO-OCT volumes were acquired. Thereby the focal plane had been set to the photoreceptor layer. Other volumes were recorded by setting the focal plane to the nerve fiber layer and inner retina (vascular layers), respectively. Finally, angiographic volumes were acquired with the AO mode. Thereby, the sampling was changed to 400 A-scans per B-scan and 4 × 300 B-scans per volume.

### Data processing

**Fig. 2:**
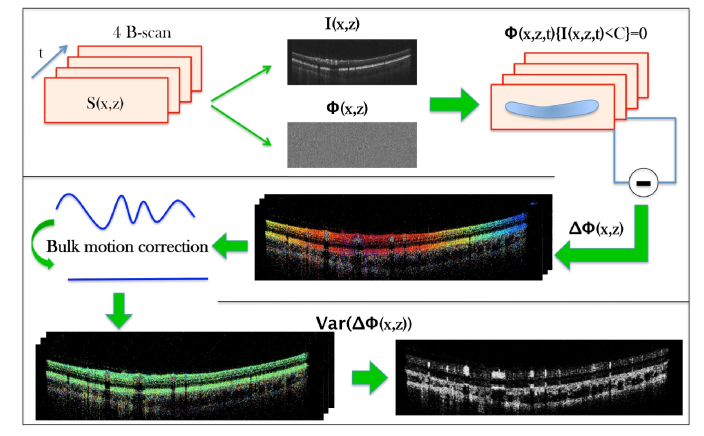
Flow diagram showing the individual post-processing steps for generating angiographic OCT data. Four B-scans that have been recorded at the same location are processed by intensity thresholding of the phase information and determining the phase variance within the set after correcting for bulk phase drifts. (t time, S recorded spectral data, I intensity, Φ Phase, C intensity noise floor ΔΦ phase difference between B-scans, Var Variance)

The used swept source provides an A-scan trigger and a k-clock signal for data acquisition. Thus, an axial depth profile can in principle be directly calculated by fast Fourier transformation (FFT) of the recorded spectrum. However, we compensated in post processing for residual dispersion mismatch prior to FFT using standard Fourier domain OCT evaluation procedure [41]. In addition, we performed spectral shaping in order to suppress the side lobes. The different post-processing steps of angiographic data evaluation are outlined in Fig. 2

. From the recorded spectral data (corresponding to 4 B-scans) OCT intensity and phase information are extracted. Data points below an intensity threshold (mean noise level extracted in the vitreous) within the OCT intensity image are excluded from further evaluation of the phase information. Axial motion between B-scans is corrected by using cross correlation of the intensity images. In a next step, the phase difference between consecutive B-scans is calculated. In order to correct for phase changes introduced by bulk motion the most frequently occurring phase difference (out of a histogram) along an A-scan is subtracted from each A-scan. Then the phase variance between the three consecutive phase difference B-scans was calculated [42]. In a final step a filter (Gaussian blur 3D with sigma 1 pixel) is applied on the resulting image and the background is subtracted considering a radius of 25 pixels. En-face angiography images are then generated by signal integration over the specified imaging depth.

## 3. Results

### Imaging of healthy volunteers

**Fig. 3:**
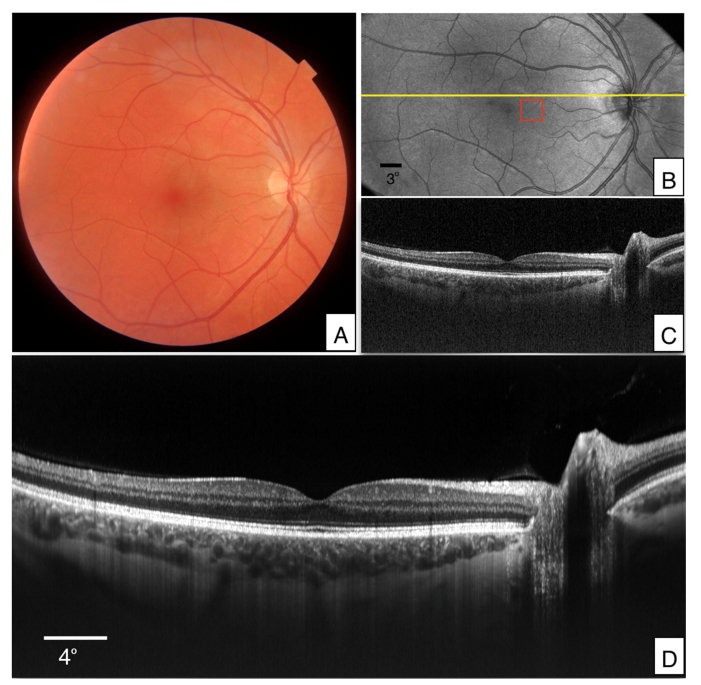
Images recorded in the right eye of a healthy volunteer (V1). A) True color fundus image acquired with a commercial instrument (45° × 45°). B) En-face image generated from volume data that was acquired with the wide FoV mode (45° × 25°) by depth integration over the entire A-scan. C) Single B-scan extracted at the location indicated with the yellow line in B). The entire data set can be viewed in Visualization 1. D) Averaged B-scan of 100 registered images that have been recorded at the location indicated with the yellow line in B). OCT B-scans are displayed on a logarithmic grey scale and the depth extension is ~4mm.

The performance of the new prototype was first tested in the right eye of a healthy, male volunteer (V1). To outline the imaged area a 45° FoV true color fundus image was acquired with a commercially available ophthalmic device (DRI OCT Triton, Topcon, Japan) for comparison (cf. Fig. 3(A)

). Representative images that were recorded with the large FoV mode of the instrument are presented in Fig. 3. Figure 3(B) shows an en-face projection that was generated from the acquired data by depth integration over the entire OCT tomogram. A corresponding B-scan recorded at a location indicated by the yellow line in Fig. 3(B) is displayed in Fig. 3(C). Due to the used wavelength region, enhanced penetration into the choroid and sclera can be seen. In order to further improve the image quality, 100 B-scans that were recorded at the same location (cf. yellow line in Fig. 3(B)) were registered to each other and averaged. The result is displayed in Fig. 3(D).

**Fig. 4:**
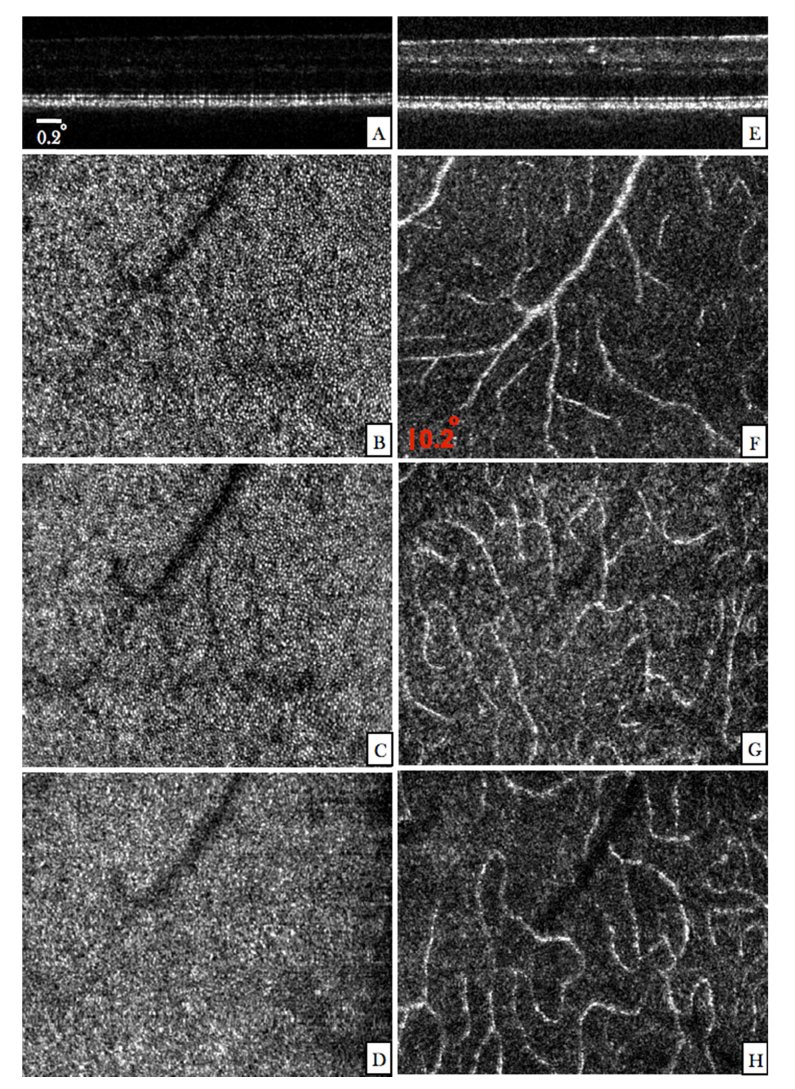
Images recorded with the AO mode of the instrument in the right eye of V1 (location is indicated by a red square in Fig. 3(B)). (A) Single B-scan extracted from a volume scan acquired with the focus set at the photoreceptor layer. (The entire data set can be viewed in Visualization 2). En-face images were generated by depth integrating the 3D data over the IS/OS (B), COST (C) and RPE (D) layers. (E) Single B-scan extracted from a volume scan acquired with the focus set at the inner retinal layers. (The entire data set can be viewed in Visualization 3). En-face images were generated from this data set by depth integration over superficial plexus (F), intermediate plexus (G), and the deep plexus (H), respectively.

In order to test the high resolution capabilities of the instrument we recorded a data set with the AO mode in the same volunteer at the location indicated by the red square in Fig. 3(B). Thereby, the focus of the system was set at the photoreceptor layer. A representative B-scan of the recorded volume is displayed in Fig. 4(A)

. Discrete spacing indicating the presence of photoreceptors can be observed at the junction between inner and outer segments of photoreceptors (IS/OS) and at the cone outer segment tips (COST). For labeling of the different outer retinal layers we refer to the work of R. Jonnal et al. [43]. The individual en-face planes of the IS/OS (Fig. 4 (B)) and COST (Fig. 4(C)) layers clearly show the cone photoreceptor mosaic. The image recorded at the retinal pigment epithelium (RPE) does not show specific structures (cf. Fig. 4(D)). As has been shown in previous work [24] several volumes need to be averaged in order to visualize RPE cells at this eccentricity from the fovea. At the same location another data set was recorded with the focus set at the inner retinal layers. In the representative B-scan (Fig. 4(E)) the brightness of the anterior layers is increased compared to the previous image (cf. Fig. 4(A)) while the discrete spacing at the photoreceptor bands is lost. The limited depth of focus associated with the high transverse resolution results in a blurred image of the photoreceptors. Figures 4(F)-(H) show corresponding en-face projection images that were generated by depth integration over the superficial, intermediate and deep plexus, respectively. Due to the high numerical aperture of the instrument, the vessels show already increased contrast in the intensity images [25, 44–46].

### OCT angiography

**Fig. 5:**
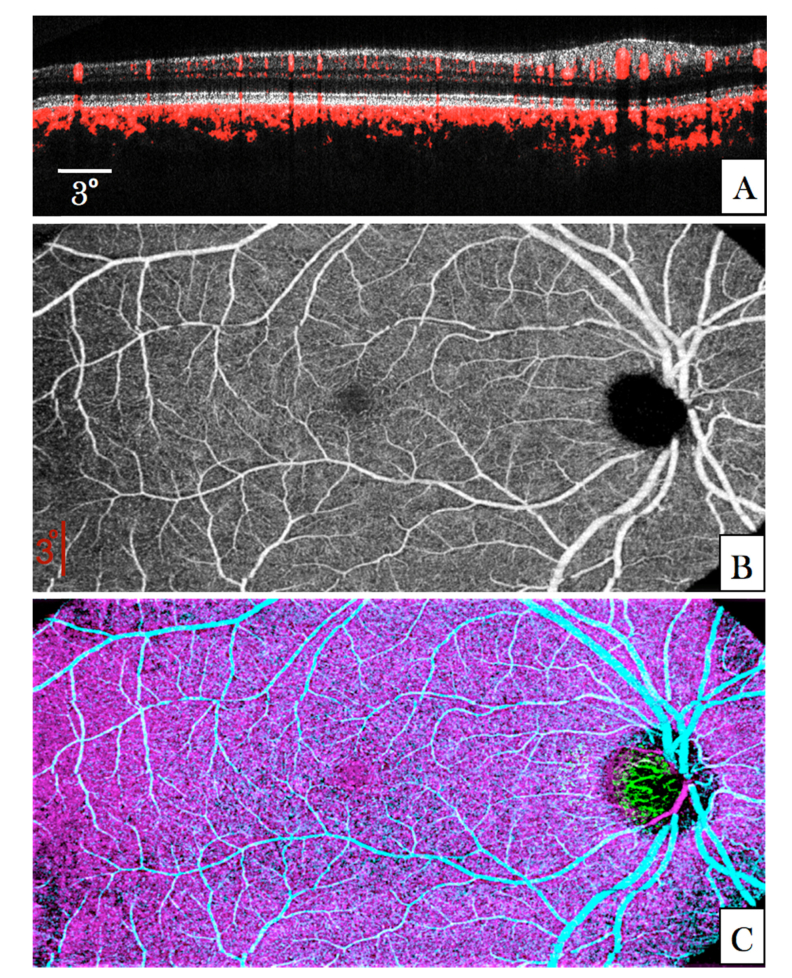
Angiographic images recorded in V2 with the large FoV mode (45° × 25°). (A) B-Scan showing the calculated phase variance within four B-scans in red overlaid to the OCT intensity image (linear gray scale, average of four images). The entire data set can be viewed in Visualization 4. (B) En-face image generated by depth integration over the inner retina. (C) Composite false color image of the phase variance in different retinal layers (inner retina (cyan), choriocapillaris (magenta) and laminar cribrosa (green)).

In a next step, the ability of the system to generate angiographic data was tested in a second healthy volunteer (V2). A representative composite large FoV B-Scan is shown in Fig. 5(A)

. The angiographic information (red) is overlaid to the OCT intensity image. Anterior vessels are visualized with improved contrast (indicated by the red color). Although the size of small capillaries is below the transverse resolution of the system and the transverse sampling for this imaging mode is rather poor, these structures introduce a phase variance that can be visualized in the OCT B-scan. In the photoreceptor band “shadowing” artefacts from larger anterior vessels can be seen (cf. red stripes in the photoreceptor band at locations below larger vessels). Otherwise these highly reflective structures do not indicate any phase changes in the image indicating a good angiographic data evaluation performance and a negligible influence of the phase changes introduced by small retinal capillaries. On the other hand, all structures posterior to the RPE show a strong phase variance. This can be attributed to phase changes introduced to the light passing through the very dense vessel network of choriocapillaris. As the transverse resolution of the large FoV mode is insufficient to separate individual vessels in this layer (inter-capillary distance ~5-20µm [47]) a rather random phase variance pattern can be observed (cf. network displayed in magenta in Fig. 5(C)).

Figure 5(B) shows an en-face image that was generated by depth integration over the inner retina of the phase variance volume. In this image larger vessels can be clearly observed. In between these, the capillary network contributes to increased phase variance although the sampling density of this imaging mode is not sufficient to resolve individual capillaries. The distance between capillaries of the inner retina is larger than the transverse resolution of the system (in contrast to vessels in the choriocapillaris). Thus the vessel network can in principle be visualized. A false color image of phase angiography at different depths (anterior plexus, choriocapillaris, and laminar cribrosa) is displayed in Fig. 5(C).

**Fig. 6:**
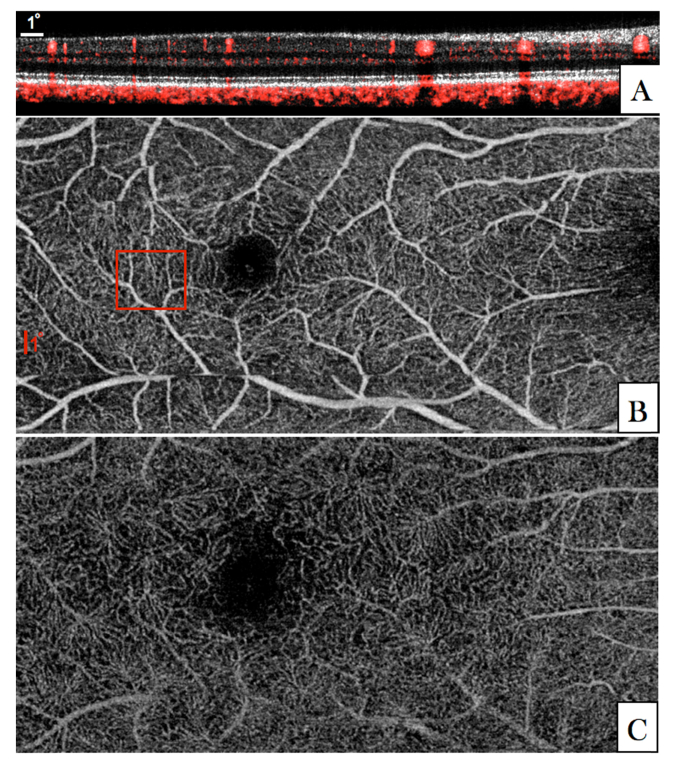
Angiographic images recorded in V2 with the medium FoV imaging mode (27°x13°). (A) Composite B-Scan showing the OCT intensity in linear grey scale and the calculated phase variance in red. The entire data set can be viewed in Visualization 5. (B) En-face image generated by depth integration of angiographic data over the superficial plexus and the deep plexus. (The red square in (B) indicates the region of interest that was imaged with the AO-OCT imaging mode).

In order to exclude the influence of the rather low transverse sampling density of the large FoV mode we recorded another data set with a medium FoV. The resulting denser sampling enables a better comparison between angiographic data that has been recorded with different transverse resolutions (with and without AO correction) later on. The acquired image data of the medium FoV mode is presented in Fig. 6

. The improved transverse sampling allows for a better visualization of small capillaries in comparison with the large FoV mode. Corresponding en-face images are shown for the superficial plexus (Fig. 6 (B)) and deep plexus (Fig. 6 (C)).

**Fig. 7:**
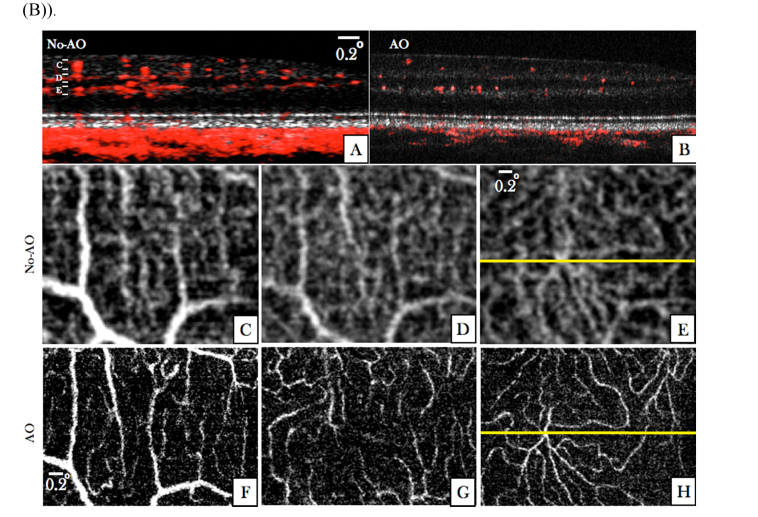
Angiographic images recorded in V2 with two different imaging modes (medium FoV mode and AO-OCT mode) at a location indicated with the red square in Fig. 6 (B). (A) Composite B-scan extracted from the data of Fig. 6 showing the OCT intensity information in gray scale and the phase variance in red. The depth extension of the different layers for en-projection are indicated by the letters C-D-E. (B) Composite B-scan recorded with the AO-OCT mode with the focus set at the inner layers. The intensity information is displayed in grey scale and the phase variance is displayed in red. (C-E) En-face phase variance images of the region of interest extracted from data of Fig. 6 of the superficial, intermediate and deep plexus, respectively. (Note that these images are enlarged views of the data presented in Fig. 6) The entire data set can be viewed in Visualization 6. (F-H) En-face images of the phase variance recorded with the AO-imaging mode by depth integration over the superficial plexus, intermediate plexus and deep plexus, respectively. The horizontal yellow line indicates the location of the B-scans.

For a detailed investigation of the influence of transverse resolution on the angiographic images, the region of interest indicated by the red square in Fig. 6 (B) was imaged with the AO imaging mode. Image results of both modes are displayed in Fig. 7

for a side by side comparison. Within the small region of interest, the different vascular layers can be extracted in both modes without the use of a specific layer segmentation algorithm. En-face images displaying superficial, intermediate and deep plexus are shown in Figs. 7 (C-E) for the medium FoV mode and in Figs. 7 (F-H) for the AO imaging mode, respectively. Without AO, shadowing artifacts can be seen in deeper layers resulting in a merging of vasculature beds. These artifacts are practically eliminated by the AO imaging mode because of the higher numerical aperture. This observation is in agreement with previous work that compared angiography of a commercial instrument with amplitude based AO-OCTA at 840nm [44]. Interestingly, some shadowing artefacts originating from larger vessels can be observed in the photoreceptor bands of the images recorded with the AO imaging mode. Because of the different algorithm for angiographic data evaluation (phase variance vs. amplitude variance) these artifacts are more pronounced in Fig. 7 than in image results of previous work [44]. This observation is in agreement with results of a detailed comparison between the different angiographic evaluation algorithms [48]. As the focus of the AO mode is set to anterior retinal layers a lower sensitivity can be observed for all structures posterior to the RPE. Thus, shadowing artifacts that are generated by transmission of the light through choriocapillaris will appear only at locations with sufficient signal to noise ratio. These locations are greatly reduced compared to the non-AO mode which results in an observation of less pronounced shadowing artifacts in this area (cf. to the red bands posterior to the RPE in Figs. 7 (A) and (B)).

**Fig. 8:**
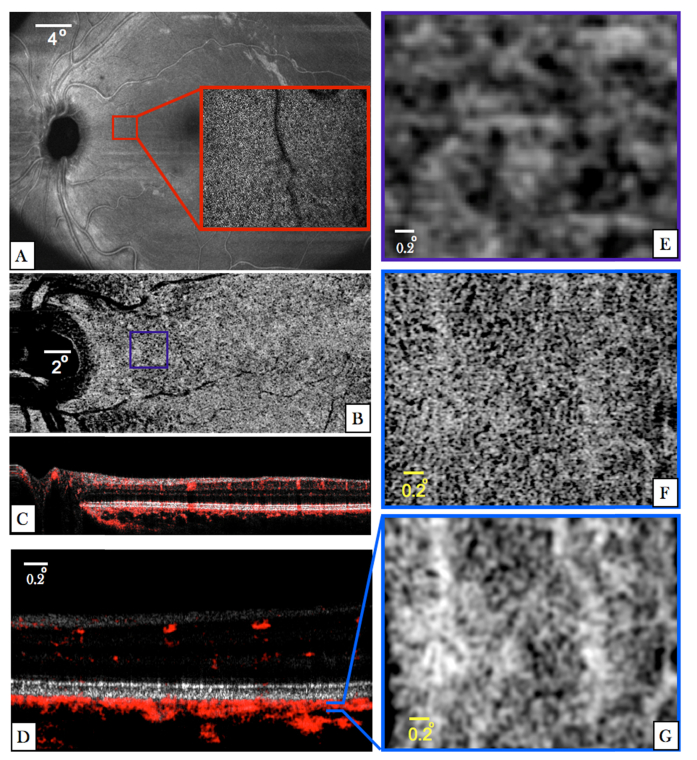
Images recorded with all three imaging modes in the left eye of V3. (A) En-face image recorded with the large FoV mode and generated by depth integration along the entire A-scan. (The red square indicates the region of interest and corresponds to 3° × 2.8° at an eccentricity of ~7.5 degrees from the fovea). The inset represents an en-face image recorded with the AO-OCT mode and was generated by depth integration over the IS/OS layer. (B) En-face image generated from the medium FoV mode by depth integration of OCTA data posterior to the RPE. (C) Composite image of OCT B-scan intensity information in linear gray scale and the phase variance in red of the medium FoV mode. (D) Composite AO-OCT B-scan with the focus set at the photoreceptor layer displaying the OCT intensity on a linear grey scale and the phase variance in red. (The entire data set can be viewed in Visualization 7) (E) Enlarged view of the region indicated by the purple square in (B). (F) En-face image generated from AO-OCT data by depth integration over a shallow slab (10 pixels) posterior to the RPE. (G) Image data from (F) after applying a Gaussian blur filter (3pixels).

In a next step we investigated the capability of the instrument to visualize choriocapillaris. For this purpose angiographic image data was recorded in the left eye of a healthy volunteer (V3). In order to select a region of interest, large FoV data was recorded. The en-face projection (depth integration over all layers) of this data set is displayed in Fig. 8(A)

. Within the region of interest (cf. red square in Fig. 8(A)) we recorded AO-OCT volumes with the focus set to the photoreceptor layer. The inset displays an en-face image that was recorded with the AO-mode and that was generated by depth integration over the IS/OS layer. Individual cone photoreceptors can be clearly observed. In addition we recorded another volume with the medium FoV mode that included the region of interest. An en-face projection (maximum intensity projection) of the OCTA data generated by depth integration over 10 pixels in depth posterior to the RPE is presented in Fig. 8(B) and shows a rather random pattern that could erroneously be interpreted as vessel network of choriocapillaris. A representative composite B-scan of this acquisition is shown in Fig. 8(C). Figure 8(D) shows a composite B-scan recorded with AO-OCT mode in the region indicated with the purple square in Fig. 8(C). For comparison with AO-OCT data an enlarged view of the region of interest is displayed in Fig. 8(E). The corresponding en-face image (cf. Fig. 8(F)) retrieved from AO image data was generated by depth integration of this data set and shows a dense capillary network. Thereby, depth integration was performed over the same shallow slab as was done for the generation of Fig. 8(E). For a better visualization of the individual vessels we applied a Gaussian blur filter (3 pixel size) over the image. The result is displayed in Fig. 8 (G). The vessel network appears very similar to results that have been recorded with other instruments [48, 49] and that have been associated with choriocapillaris. However, with our instrument a single AO-OCTA volume acquisition was sufficient to generate this image and averaging over several volumes was unnecessary. We want to emphasize here the different appearance of the vessel network in our images that have been recorded with and without AO.

### Patient imaging

**Fig. 9:**
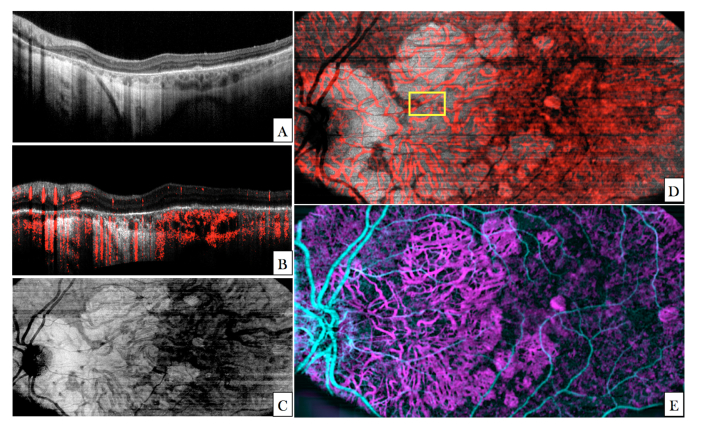
Images recorded with the large FoV mode in a patient with geographic atrophy. (A) Central B-scan showing the atrophic zone on the left half of the image. (B) Flattened central B-scan with angiographic information overlaid in red. (C) En-face image generated by depth integration of the volume data posterior to RPE/Bruch’s membrane. (D) The same en-face image as shown in (C) with the angiographic information overlaid in red. (The yellow square indicates the region of interest that has been imaged with AO-OCT- cf. Fig. 10). (E) Composite image generated by angiographic evaluation of the data visualizing vasculature of the inner retina (cyan) and choroid (magenta).

Representative images recorded with the large FoV mode of the instrument in a patient presenting with an advanced stage of AMD are shown in Fig. 9

. Extended areas of geographic atrophy (GA) can be seen in the left half of the large FoV B-scan (Fig. 9 (A)). In these areas the RPE and photoreceptors are degenerated which leads to an enhanced penetration into the choroid and sclera. The corresponding en-face projection image is displayed in Fig. 9 (C). The composite B-scan (Fig. 9(B)) shows the flattened intensity image and the angiographic information overlaid in red. Larger vessels are clearly visualized in the anterior retina. Posterior to the RPE/Bruch’s membrane choroidal vessels are visualized in the angiographic image mainly in regions with RPE atrophy (left hand side). In regions with intact RPE and choriocapillaris, all posterior structures with the exception of larger choroidal vessels are visible in the angiographic image (cf. right hand side in Fig. 9(B)). The en-face intensity projection over the choroid (located between RPE/Bruch’s membrane and sclera) clearly shows the larger choroidal vasculature as hypo reflective structures throughout the image. In the atrophic zone an enhanced signal (in comparison with the non-atrophic areas) in the tissue surrounding these vessels can be observed. An identical en-face projection of the angiographic image data is displayed in red overlaid to the intensity image in Fig. 9(D)). In the atrophic area the vessels visualized by angiographic evaluation clearly match the hypo-reflective structures of the intensity image. However, in the non-atrophic region a vessel-like pattern can be seen in the angiographic image that does not correspond to the hypo-reflective structures of the intensity image. Figure 9(E) shows the entire angiographic information of the volume with the different imaging depths encoded in false color (inner retina: cyan, choroid: magenta).

**Fig. 10:**
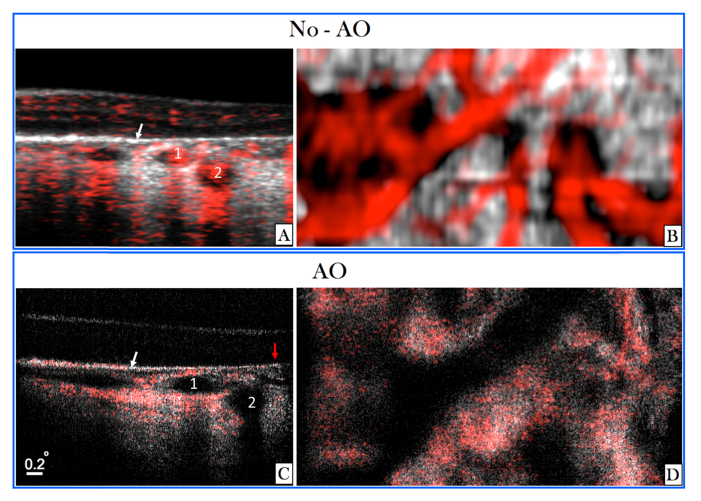
Images recorded in a patient with geographic atrophy in the region of interest indicated in Fig. 9(D)). A) Composite B-scan recorded with the large FoV mode showing OCT intensity and angiographic information overlaid in red (enlarged view of the region of interest approximately corresponding to the location displayed in C). The white arrow indicates the border between atrophic and non-atrophic region. The numbers 1 and 2 indicate two vessels. B) Corresponding en-face projection over the depth extension of choroid of the large FoV data. C) Composite B-scan recorded with the AO-OCT imaging mode. The numbers 1 and 2 indicate the same two vessels that are displayed in A). The red arrow indicates a location without larger choroidal vessels. D) En-face projection of AO-OCT data (over the depth extension of choroid).

AO-OCT data was acquired at a location that is indicated with the yellow square in Fig. 9 (D). The region of interest contains atrophic and non-atrophic areas. A side by side comparison of images recorded in this region of interest with and without AO is shown in Fig. 10

. In the central B-scan (cf. Fig. 10(A)) the atrophic region is located on the right hand side of the image. The approximate border between the two areas is indicated with a white arrow. Angiographic information can be seen mainly in the atrophic region within the hypo-reflective structures of the choroid (cf. numbers 1 and 2 in the image). Below these vessels shadowing artifacts can be seen in the surrounding tissue. In the region with intact RPE (left hand side), hyper-reflective structures of the choroid additionally show phase variance which can be explained by transmission of the light through choriocapillaris and the generation of a corresponding shadowing artefact. Much finer details of the choroidal structures can be seen in the B-scan that was recorded with the AO-mode. Due to the lower sensitivity of this imaging mode, the phase variance could not be calculated within the hypo-reflective choroidal vessels. Thus, these appear dark in the image. However, because of the transmission of the light through these vessels, phase variance can be observed mainly below the vessels. At the right margin of the image (indicated with a red arrow)) negligible phase variance can be seen throughout imaging depth indicating that at this location no larger choroidal vessels are present. In the region located left to the white arrow, phase variance can be seen within the RPE/Bruch’s membrane complex. This might be caused by the presence of choriocapillaris.

## 4. Discussion

In this paper we presented a new compact OCT prototype that is capable of providing large FoV images with standard OCT resolution as well as small FoV images with high resolution and AO correction (cf. Figs 3 and 4). Switching between imaging modes can be easily and fast performed via flip mirrors in the sample and reference arms, respectively. Thus, regions of interest that need to be viewed in more detail can be localized using the large FoV mode during an imaging session. These regions can then be imaged with the AO imaging mode which can be regarded as an optical zooming function of the system. With this functionality the proposed prototype enables new perspectives for patient imaging. It addresses the inherent problem of small FoVs which is common to many AO assisted instruments. Instead of stitching a multitude of recorded AO-data sets for generating a larger FoV which is very time consuming and not clinically feasible, only regions of interest that can be pre-selected in the large FoV mode will be imaged.

One drawback of our instrument is that for the AO mode the fixation target has to be moved in order to localize the region of interest. This requires compliance and certain fixation capabilities of the subject which is not optimal. However, we found that the latter is also an essential requirement for minimizing motion artefacts and that the implementation of steering mirrors [50] would significantly increase the instrument size.

We want to emphasize here that the presented concept for large and small field of view imaging is different from what has been presented, recently [31, 51]. Due to the different optical paths of the AO and large FoV imaging modes the diameter of the imaging beam can be varied which results in a moderate transverse resolution of the large FoV mode (0.6 mm beam diameter and 30µm transverse resolution). Thereby, aberrations introduced by the eye (including defocus) will have a low impact on this imaging mode. On the other hand, a larger imaging beam diameter (6mm) can be used for the AO-mode which results in high transverse resolution (3µm) of this imaging mode. In contrast to that, the compact system introduced by J. Polans et al. [31] used varying scanning angles in order to obtain different FoVs. Thus, the instrument was designed with an imaging beam diameter of 3mm which represents a compromise between high transverse resolution and low sensitivity to wavefront aberrations. (The latter is essential for the large FoV mode.) A concept that has also been used for visualizing cone photoreceptors without the need of AO correction [11]. Another difference of the instruments is the AO correction. Here we use wavefront sensing based AO correction that can be operated at 10Hz loop time while the instrument in Ref [31]. relies on a cheaper sensor less AO correction approach that usually is rather slow (several seconds are needed for the correction).

The OCT engine itself is based on swept source technology at 1060nm and uses an akinetic light source. For a fixed imaging beam diameter, the longer wavelength region results in a lower transverse resolution compared to imaging at 850nm or 790nm. In order to compensate for that the diameter of the AO mode has been increased from 5mm to 6mm in comparison with one of our previous instruments [30, 44]. As has already been demonstrated for standard OCT imaging [52–54] and for OCTA [55] the shift of the wavelength region results in an enhanced penetration into the choroid and sclera and may provide better image quality in the presence of ocular turbidity. Thus, the instrument may provide a wider range of clinical applications for both the large FoV and AO imaging modes compared to an instrument operating in the shorter wavelength range as deeper retinal structures may be visualized.

One drawback of the used wavelength region is the limited axial resolution that can be achieved. Even in the case of a large sweeping range (or a broad spectral width of the light source in the case of spectral domain OCT), the water absorption by the ocular media will limit the axial resolution in the retina [56]. In our case the sweeping range of 60nm at 200kHz A-scan rate is determined by the used light source. The resulting axial resolution of 7.3µm (in tissue) is at the border of what can be regarded as still acceptable because the separation of retinal layers that are close together is still visible but not very pronounced. As an example of neighboring retinal layers we want to emphasize COST and RPE (cf. Fig. 4(A)). Although in the B-scan these layers can be hardly distinguished, both layers seem to be separable in the corresponding en-face images (cf. Figs. 4(C) and 4(D)). Nevertheless, a higher axial resolution would be preferable as the axial speckle size will be smaller and a clearer separation between these layers will be possible, especially within the B-scans. A higher axial resolution might also be a crucial issue for visualization of RPE cells as the corresponding mosaic appears only within a very thin layer [24]. In principle, the system can be upgraded with a swept source that provides a larger sweeping range. Newer versions of the 1060 akinetic source could be operating with broader tuning bandwidth at 200kHz.

The current imaging speed of the system may be improved as well. For the large FoV and AO imaging modes, the sampling density is already at the limit. The proposed sampling density was chosen in order to provide a compromise between minimization of acquisition time (and corresponding motion artefacts) and maximizing field of view. However, as can be seen in the angiographic evaluation of the large FoV data, a higher density is required in order to fully exploit the visualization capability of the system in the presence of small retinal capillaries (compare Figs. 5(B) and 6(B)). Similar, a higher sampling density of the AO images will certainly improve the visibility of individual cells such as cone photoreceptors and therefore facilitate a quantitative evaluation (e.g. cone counting) of the data. We want to emphasize here that a reduction of the field of view will not help to solve this problem because in this case motion artefacts will be more pronounced which leads to distorted image data. Thus, the only solution will be the implementation of a faster light source (akinetic light source or Fourier domain locked laser [57]) at the cost of a lower sensitivity.

A clear benefit of the used swept source in contrast to spectral domain OCT systems is the preservation of sensitivity within 4mm imaging depth. This greatly elevates the need for precise subject alignment (i.e. imaging close to zero delay) as axial motion does not severely influence the signal intensity. In addition, imaging with large FoV or in the presence of pathologies with extended structures such as RPE detachments is facilitated.

The excellent phase stability between A-scans which is comparable to spectral domain systems is certainly an advantage of the current instrument. Thus, angiographic data can be calculated using the phase information (without the need of additional efforts for phase stabilization) and a multitude of different algorithms [58] can be applied to the data. In order to demonstrate the phase stability we restricted the evaluation to the calculation of the phase variance which is able to detect even very small motions. In addition, the phase stability allows for Doppler evaluation of the data. We want to point out here that the evaluation procedure is different compared to our previous work [44] where the amplitude variance was used to generate AO-OCTA images. Associated with this are subtle differences in the corresponding angiographic images. A closer look at the AO-OCT B-scans reveals that in the case of phase variance, despite the high numerical aperture, shadowing artefacts are generated in deeper layers (e.g. photoreceptor layer) by larger vessels (cf. Fig. 7(B)). In the case of amplitude variance these artifacts are less pronounced (cf. Figs. 5(G) and (H) in Ref [44].).

The projection artifacts are even more pronounced in the large FoV mode, as can be seen in Figs. 5(A) and 6(C). The artifacts originate from motion within vessels that introduces a subsequent change in the phase of the light that traverses it. Thus, all layers below the vessel will show a similar phase variance even if these represent static tissue. This can be clearly seen in Figs. 6(A) and 7(A) by the high phase variance (indicated by the red color) below larger vessels within the photoreceptor layer.

Posterior to the RPE all structures show high phase variance. There are some aspects that indicate that this mainly originates from transmission of the light through choriocapillaris. First of all, it needs to be considered that phase variance can only be meaningfully calculated in areas with sufficient signal intensity. Areas with no or low signal (vitreous, outer nuclear layer, choroidal vessels) are commonly excluded from the phase analysis (only pixels above a certain intensity threshold are used) and will therefore not contribute to the angiographic image. The consequences of this evaluation procedure can be seen in the images showing the geographic atrophy (cf. Fig. 9). In the atrophic regions (no presence of RPE) the signal from deeper layers is stronger than in the non-atrophic areas. Thus, a sufficiently high signal (above the intensity threshold) from the choroidal vessels is observed (cf. left hand side of Fig. 9(B)) and these data points are included in the evaluation. Due to the high flow speed in these vessels a large phase variance is introduced. In addition, choriocapillaris seems to be degenerated in these areas. Thus, high phase variance observed within these posterior layers originates solely from choroidal vessels. Both, higher signal intensity and degeneration of choriocapillaris result in a high contrast angiographic image of choroidal vessels in the geographic atrophy area that completely corresponds to the hypo-reflective vessel structure of the intensity en-face projection.

On the other hand, in areas with intact RPE and choriocapillaris, the signal within the choroidal vessels is too low to fully contribute to the angiographic image. In addition shadowing artifacts are generated by transmission of light through choriocapillaris. Thus, choroidal vessels appear dark in the angiographic image while the surrounding static tissue shows high phase variance. As a consequence the angiographic image in this area does not represent the vessel structure of the choroid.

In a similar way, the appearance of the angiographic images recorded with the AO imaging mode can be explained. The signal from choriocapillaris is sufficiently large to contribute to the angiographic image and the vessel structure of this layer can be revealed (cf. Fig. 8(G)). However, because of the lower sensitivity of this imaging mode compared to the large FoV mode, choroidal vessels show weak signal intensity and are partly excluded from the analysis. Together with the projection artifacts caused by choriocapillaris this produces an angiographic image that shows a mixture of static and dynamic tissue and a corresponding interpretation of the images is difficult. This analysis of AO-OCTA data deviates from interpretations that have been presented in previous work [49].

Finally, we want to emphasize the difference between angiographic images of choriocapillaris that were recorded with the different imaging modes (cf. Figs. 8(E) and 8(G)) and the general problematic of angiographic imaging of choroidal layers. Although, the large FoV image shows a pattern that is similar to a capillary network, the size and distribution does not resemble the pattern that can be observed with the AO imaging mode. The reason for this is that the transverse resolution of the large FoV mode (~30µm) is insufficient to separate the densely packed vessel network of choriocapillaris. In fact, the large FoV image only indicates the presence of flow within the layer and smaller structures (dark and bright patterns) do not represent meaningful information. Thus, a careful interpretation of angiographic images of choriocapillaris recorded with standard OCT resolution is essential. For visualizing the capillary network of choriocapillaris a higher transverse resolution as provided by research grade instruments [48] or AO-OCT is essential [49]. As for angiographic imaging of deeper layers of the choroid, the data will always be affected by projection artifacts introduced by passing of the light through an intact choriocapillaris. Thus, the interpretation of this data needs to be done carefully.

## 5. Conclusion

In conclusion we presented a compact, clinical usable SS-OCT instrument that supports different imaging modes. The large field of view mode generates OCT data with standard resolution and can be used, apart from routine diagnostic imaging, for determination of regions of interest. These regions can then be investigated in great detail using the AO-mode of the instrument. Both imaging modes provide angiographic information on the vascular structure of the retina by exploiting the high phase stability of the used akinetic swept source. The large FoV mode only indicates the presence of choriocapillaris while the AO imaging mode is essential for resolving individual vessels within this layer. Initial performance tests in healthy volunteers and patients are promising and the proposed method might evolve into a valuable tool for improved diagnosis and treatment control of retinal diseases.
